# Disproportionality analysis of biliary adverse events associated with fibrates using the JADER and FAERS databases

**DOI:** 10.3389/fphar.2025.1700589

**Published:** 2025-11-24

**Authors:** Satoko Watanabe, Kyosuke Nagura, Naoto Okada, Taro Watanabe, Hidenori Sagara

**Affiliations:** 1 Division of Medical Safety Science, Faculty of Pharmaceutical Sciences, Sanyo-Onoda City University, Sanyo-Onoda, Japan; 2 Pharmacy Department, Yamaguchi University Hospital, Ube, Japan; 3 Department of Pharmacy, Yamaguchi Prefectural Grand Medical Center, Hofu, Japan; 4 Division of Medical Safety Science, Graduate School of Pharmaceutical Sciences, Sanyo-Onoda City University, Sanyo-Onoda, Japan

**Keywords:** fibrates, pemafibrate, biliary adverse events, pharmacovigilance, disproportionality analysis, time-to-onset analysis, JADER, FAERS

## Abstract

**Introduction:**

Fibrates are effective triglyceride-lowering drugs, but they may affect bile acid metabolism, raising concerns about biliary adverse drug events (ADEs).

**Objective:**

In this study, we used spontaneous reporting system databases to evaluate the association between fibrates and biliary ADEs. This study has been reported in accordance with the Reporting of a Disproportionality Analysis for Drug Safety Signal Detection Using Individual Case Safety Reports in PharmacoVigilance guidelines.

**Methods:**

We used data from the Japanese Adverse Drug–Event Report (JADER) and the U.S. Food and Drug Administration Adverse Event Reporting System (FAERS) databases. The signal detection metrics employed were reporting odds ratio (ROR), proportional reporting ratio, Bayesian confidence propagation neural network, and Gamma–Poisson Shrinker. We also conducted stratified disproportionality and time-to-onset analyses.

**Results:**

We identified 58 and 260 unique individual case safety reports from the JADER and FAERS databases, respectively. Primary disproportionality analysis of all fibrates in the JADER dataset revealed an ROR of 3.74 [2.88–4.85]. All other signal detection metrics also exhibited statistically significant associations. In the stratified disproportionality analysis, pemafibrate showed significant signals across all strata, confirming the robustness of the signal. In the Weibull analysis for pemafibrate, the shape parameter (β) was 1.59 [1.17–2.56], indicating an increasing trend in ADE reporting with continued pemafibrate use.

**Conclusion:**

A significant signal for biliary ADEs was detected for fibrates in both databases, with a particularly consistent association for pemafibrate. Regular hepatobiliary monitoring and individualized patient management are recommended.

## 1 Introduction

Fibrates are widely used to lower triglyceride levels. Their mechanism of action involves the activation of peroxisome proliferator–activated receptors (PPARs), a family of transcription factors in the nuclear hormone receptor superfamily (Staels et al., 1998). Of the three PPAR subtypes (α, β/δ, γ), PPARα primarily mediates the lowering of triglyceride levels (Staels et al., 1998).

PPARα suppresses the activity of cholesterol 7α-hydroxylase (CYP7A1) and sterol 27-hydroxylase (CYP27A1), the rate-limiting enzymes in bile acid synthesis (Post et al., 2001; Ghonem et al., 2015), leading to a decrease in bile acid production and the likely development of biliary tract–related adverse effects, including cholelithiasis (Post et al., 2001). A case series suggested an association between fibrates use and cholelithiasis (Caroli-Bosc et al., 2001). A 52-week Phase III trial of pemafibrate reported a cholelithiasis incidence of 5.3% (Kowa Co.ltd., 2024). Pemafibrate is a selective PPARα modulator approved in Japan in 2017 (Kowa Co.ltd., 2024).

Evidence linking fibrates to biliary adverse drug events (ADEs) remains limited. Clinical trials are conducted under controlled conditions and may not reflect real-world practice (Singh and Loke, 2012). Post-marketing, high-quality prospective studies on biliary ADEs are scarce, particularly in older adults and individuals on multiple medications (Cohen et al., 2024). Moreover, the use of pemafibrate is currently confined to a few countries, including Japan, Singapore, and Thailand, resulting in a paucity of data on adverse events.

Spontaneous reporting systems (SRSs) are increasingly being used in drug safety evaluation (Alomar et al., 2020). Understanding drug safety warrants multiple data sources in addition to clinical trials (Singh and Loke, 2012). SRS databases provide complementary information, especially for drugs with limited use, such as pemafibrate (Noguchi et al., 2021). The Japanese Adverse Drug–Event Report (JADER) and the Food and Drug Administration Adverse Event Reporting System (FAERS) are two of the prominent SRS databases available.

In this hypothesis-generating study, we examined the association between fibrates and biliary tract ADEs using JADER and FAERS. The study aimed to detect potential safety signals and describe disproportionality and reporting patterns to inform future pharmacoepidemiologic research.

## 2 Materials and methods

### 2.1 Database sources

Individual case safety reports (ICSRs) were obtained from the JADER database, maintained by the Pharmaceuticals and Medical Devices Agency (PMDA). Reports were also obtained from the FAERS database, maintained by the U.S. Food and Drug Administration (FDA). Data were downloaded from the official PMDA and FDA websites. The data snapshots were as follows: JADER (downloaded 6 June 2025) and FAERS (quarterly files through 2024 Q4; compiled dataset last updated on 16 June 2025). The JADER dataset covered April 2004 to May 2025. The FAERS dataset covered Q1 2014 to Q4 2024.

**FIGURE 1:**
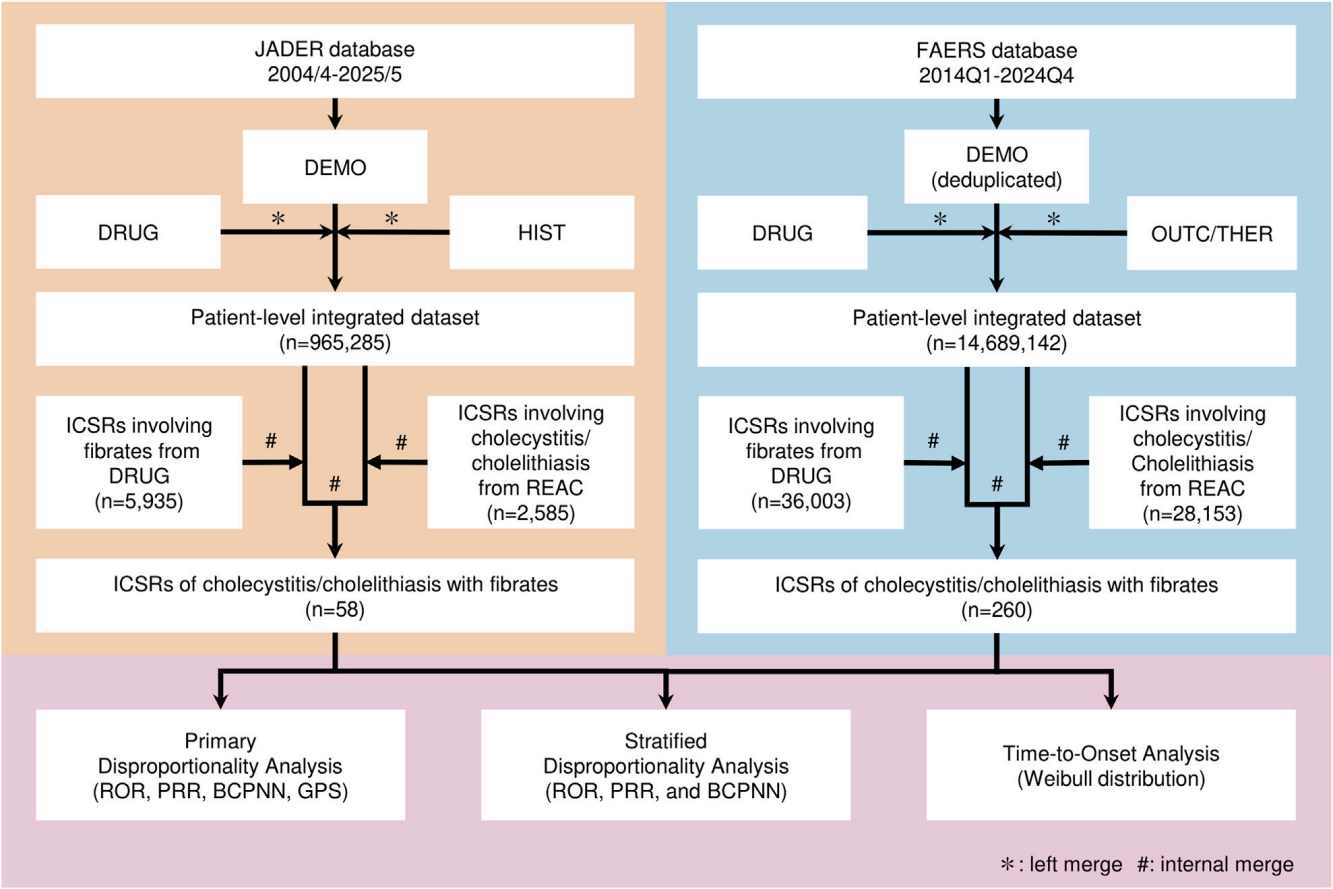
Flowchart of Data Analysis of JADER and FAERS Data. This flowchart shows the series of steps involved in data preprocessing, case selection, and signal detection in the JADER and FAERS datasets. JADER, Japanese Adverse Drug–Event Report database; FAERS, Food and Drug Administration Adverse Event Reporting System; Q1, first quarter; Q4, fourth quarter; ICSRs, individual case safety reports; ROR, reporting odds ratio; PRR, proportional reporting ratio; BCPNN, Bayesian confidence propagation neural network; GPS, Gamma–Poisson Shrinker.

In the JADER dataset, the DRUG and HIST tables were left-merged with the DEMO table to create a patient-level dataset (Figure 1). In the FAERS dataset, duplicate reports were removed by keeping the record with the highest case version number in the DEMO table. The DRUG and DEMO tables were then left-merged to create a patient-level dataset. The OUTC table was merged for disproportionality analyses, and the THER table was merged for time-to-onset (TTO) analysis.

### 2.2 Identification of individual case safety reports involving fibrates and biliary adverse drug events

To identify fibrate-related reports in the JADER dataset, we used the Japanese generic names for pemafibrate, fenofibrate, and bezafibrate. In the FAERS dataset, we searched for “PEMAFIBRATE,” “FENOFIBRATE,” “FENOFIBRIC ACID,” “CHOLINE FENOFIBRATE,” and “BEZAFIBRATE,” standardizing the spelling variations of “Fenofibrate.” We included all drug–event pairs in which a fibrate was listed in any drug role (e.g., suspect, concomitant, or interacting) to avoid reporting bias. We also conducted a sensitivity analysis in which the disproportionality analysis was limited to primary suspect drugs. We defined a drug–event pair as a unique combination of one drug and one adverse event. When a single ICSR contained multiple drugs or events, all possible combinations of these were counted as separate pairs. Because a single ICSR may include multiple fibrates (e.g., concomitant use or drug switching), agent-level counts are not mutually exclusive. The overall total for “all fibrates” reflects unique ICSRs. Consequently, the sum of agent-level counts may not equal the overall total.

Biliary events were identified using the Japanese translation of the Medical Dictionary for Regulatory Activities, version 28.0. Fourteen preferred terms under the high-level term “Cholecystitis and cholelithiasis” (10008616) were included (Supplementary Table 1).

### 2.3 Primary disproportionality analysis

For signal detection, we constructed a 2 × 2 contingency table (Table 1).

**TABLE 1:** 2 × 2 contingency table.

	Target adverse drug–event	Other adverse drug events	Sums
Fibrates	n11	n12	n1+
Other drugs	n21	n22	n2+
Sums	n+1	n+2	n++

A 2 × 2 contingency table was constructed using fibrates as the drugs of interest and biliary ADEs (defined by 14 PTs under the HLT “Cholecystitis and cholelithiasis”) as the events of interest.

n11: drug–event pairs that involved fibrate use and indicated cholecystitis or cholelithiasis.

n12: drug–event pairs that involved fibrate use and did not indicate cholecystitis or cholelithiasis.

n21: drug–event pairs that did not involve fibrate use but indicated cholecystitis or cholelithiasis.

n22: drug–event pairs that did not involve fibrate use and did not indicate cholecystitis or cholelithiasis.

PTs, Preferred Terms; HLT, high-level term.

n11 represented drug–event pairs involving fibrates and cholecystitis or cholelithiasis; n12 represented drug–event pairs involving fibrates without these events; n21 represented drug–event pairs involving these events without fibrates; and n22 represented drug–event pairs involving neither. We calculated the reporting odds ratio (ROR) and proportional reporting ratio (PRR). We also calculated the information component (IC) using the Bayesian confidence propagation neural network and the empirical Bayes geometric mean (EBGM) using the Gamma–Poisson Shrinker estimator.

ROR, a sensitive screening metric, is a standard metric in JADER (Sakaeda et al., 2013). A signal was considered present when n11 ≥ 3 and ROR025, the lower bound of the 95% confidence interval (95% CI), exceeded 1. P values were obtained using Fisher’s exact test.

PRR compares the event proportion for a drug against all other drugs, and it follows the implementation of the Medicines and Healthcare products Regulatory Agency (Evans et al., 2001). PRR025, the lower bound of the 95% CI, was used to more strictly evaluate drugs that had passed ROR-based initial screening. While this higher threshold can reduce false positives, it may also increase the risk of missing important signals (Harpaz et al., 2013). A signal was considered present when n11 ≥ 3, PRR025 > 2, and the chi-square (χ^2^) statistic >4.

The Bayesian confidence propagation neural network is a Bayesian approach that stabilizes estimates for sparse data (Bate et al., 1998; Orre et al., 2000). A signal was considered present when IC025, the lower bound of the 95% credibility interval of IC, exceeded 0.

The Gamma–Poisson Shrinker estimator uses empirical Bayes shrinkage with stratification (Dumouchel, 1999; Szarfman et al., 2002; 2004). In the JADER dataset, EBGM was estimated using a stratified model. Reports were stratified by sex (male/female) and age group (≥60 vs. 20–50 years). Expected counts within each stratum were computed under the independence assumption and pooled across strata to obtain the EBGM and its 90% CI. A signal was considered present when EBGM05, the lower bound of the 90% CI, was equal to or greater than 2.0. EBGM was not computed for FAERS data because stratification was not performed.

The formulas used, confidence intervals computed, and parameter settings applied are provided in Supplementary Methods 1.

### 2.4 Stratified disproportionality analysis

Stratified analysis was performed for sex, age, and body mass index (BMI). Sex was categorized as male or female, age as ≥60 years or 20–50 years, and BMI as ≥25 or <25. For calculating BMI, the median value for each height and weight category was used, given that JADER records these in 10-cm and 10-kg increments. Reports with missing data for the target stratum were excluded.

Stratified analysis was not performed on FAERS data. Pemafibrate use is concentrated in Japan and a few other countries; therefore, only a few reports were available on the FAERS database (Arai et al., 2024), precluding BMI calculation.

### 2.5 Time-to-onset analysis

TTO analysis was limited to pemafibrate data from the JADER dataset with complete therapy-start and event onset dates (N = 17); reports with incomplete or ambiguous dates were excluded. TTO was defined as the number of days from drug initiation to event onset, with 1 day added to avoid zero-day values when the event occurred on the start date (Ando et al., 2019). The descriptive statistics for TTO analysis included median, first quartile, third quartile, maximum, and minimum. A box-and-whisker plot was generated.

For Weibull analysis, the shape (β) and scale (α) parameters were estimated using maximum likelihood estimation, and the Weibull curve was plotted. Because only 17 reports contained complete date information, 95% CIs for α and β were calculated through bootstrapping (Estadística et al., 2018). β was interpreted as wear-out if the lower bound >1, random if the interval included 1, and early failure if the upper bound <1 (Leroy et al., 2014).

### 2.6 Analytical tools and Software environment

Analyses were performed using the MSIP platform (version 1.10.1; NTT DATA Mathematical Systems Inc., Tokyo, Japan) and the Python programming language (version 3.13). ChatGPT (OpenAI, GPT-5, https://chat.openai.com/) was used to support the generation of some Python prompts for figure preparation; all outputs were reviewed and verified by the authors. This study has been reported in accordance with the Reporting of a Disproportionality Analysis for Drug Safety Signal Detection Using ICSRs in PharmacoVigilance guidelines (Fusaroli et al., 2024).

The requirement for an institutional ethics review was waived because the study used publicly available, de-identified data (see Ethics Statement).

### 2.7 Ethics statement

This study analyzed publicly available, de-identified reports from the JADER and FAERS databases; therefore, approval from the institutional review board was not required. For the clinical vignette, all potentially identifiable information was removed, and patient consent for publication was obtained.

## 3 Results

### 3.1 Basic characteristics of the individual case safety reports

In the JADER dataset, the proportion of male ICSRs was 67.9% for pemafibrate, 70.0% for fenofibrate, and 69.2% for bezafibrate (Table 2). ICSRs from individuals aged ≥60 years accounted for 67.9% of the pemafibrate reports, 80.0% of the fenofibrate reports, and 61.5% of the bezafibrate reports. Among the fenofibrate ICSRs, only one was submitted by a consumer; all other reports, including those for pemafibrate and bezafibrate, came from healthcare professionals.

**TABLE 2:** Basic characteristics of ICSRs in the JADER database.

Variables	Pemafibrate	Fenofibrate	Bezafibrate
Sex			
Male	19 [67.9]	14 [70.0]	9 [69.2]
Female	9 [32.1]	6 [30.0]	4 [30.8]
Not specified	0 [0.0]	0 [0.0]	0 [0.0]
Age			
20–50 years	9 [32.1]	4 [20.0]	5 [38.5]
≥60 years	19 [67.9]	16 [80.0]	8 [61.5]
Reporter			
Healthcare professional	28 [100.0]	19 [95.0]	13 [100.0]
Consumer	0 [0.0]	1 [5.0]	0 [0.0]
Unknown	0 [0.0]	0 [0.0]	0 [0.0]
Year			
2004	–	–	–
2005	–	1 [5.0]	1 [7.7]
2006	–	–	1 [7.7]
2007	–	1 [5.0]	–
2008	–	–	1 [7.7]
2009	–	–	–
2010	–	–	1 [7.7]
2011	–	–	–
2012	–	2 [10.0]	–
2013	–	–	1 [7.7]
2014	–	1 [5.0]	–
2015	–	–	1 [7.7]
2016	–	1 [5.0]	1 [7.7]
2017	–	2 [10.0]	1 [7.7]
2018	–	2 [10.0]	4 [30.8]
2019	6 [21.4]	–	–
2020	2 [7.1]	–	1 [7.7]
2021	7 [25.0]	2 [10.0]	–
2022	5 [17.9]	1 [5.0]	–
2023	3 [10.7]	6 [30.0]	–
2024	5 [17.9]	1 [5.0]	–

This table summarizes the characteristics of ICSRs related to biliary ADEs for each fibrate in the JADER database. Per-drug counts are ICSRs and are not mutually exclusive. The characteristics include patient sex, age, reporter occupation, and year of report. Numbers in parentheses indicate the proportion of reports within each category.

ICSR, individual case safety report; JADER, Japanese Adverse Drug–Event Report; ADEs, adverse drug events.

### 3.2 Primary disproportionality analysis

**FIGURE 2:**
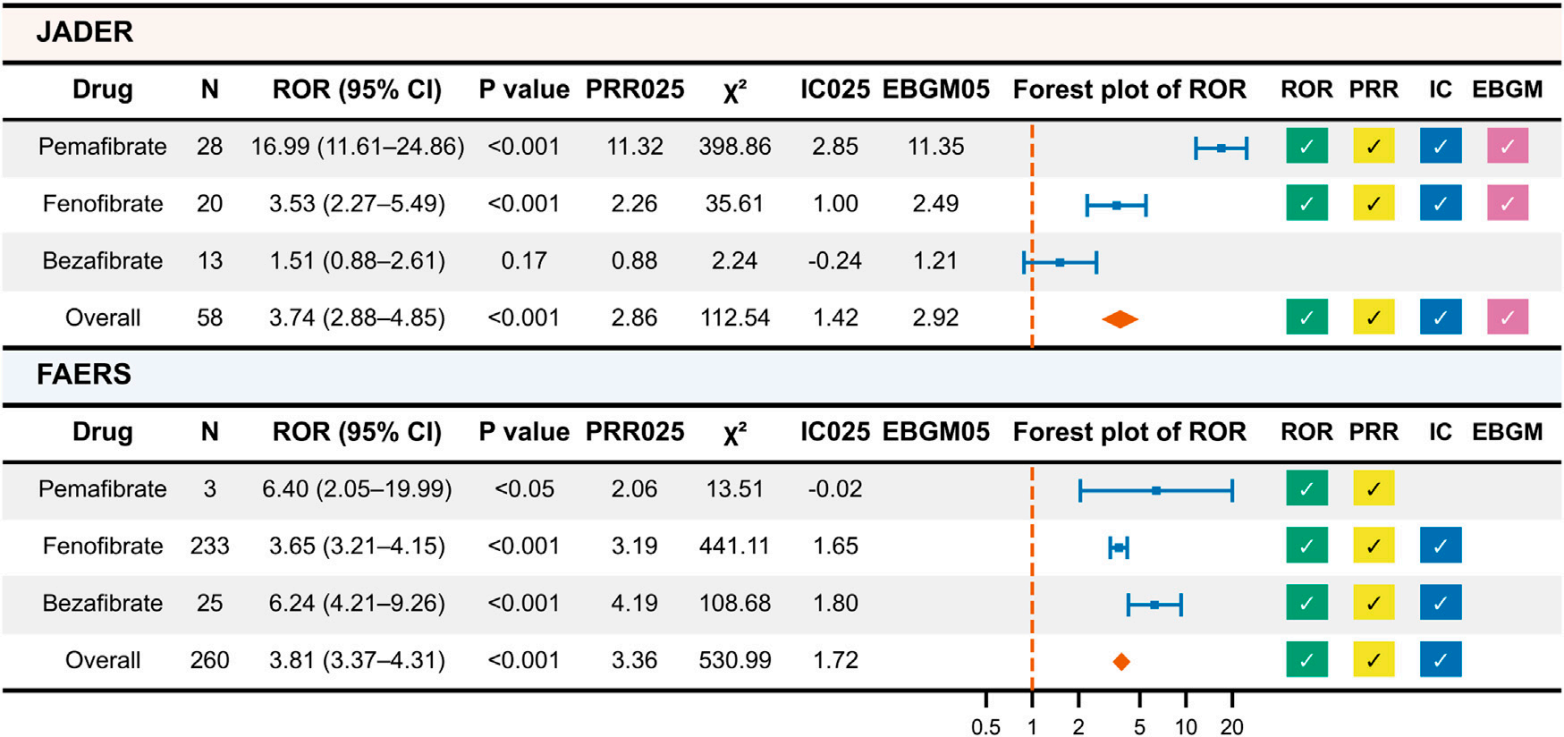
Results of Primary Disproportionality Analysis of JADER and FAERS Data. This figure presents the results of the primary disproportionality analysis conducted on the JADER and FAERS datasets. A check mark indicates a statistically significant signal. N (per-drug) = drug–event pairs. “Overall” = unique ICSRs. Per-drug counts are not mutually exclusive; therefore the total may not equal the sum across drugs. IC, information component; EBGM, empirical Bayes geometric mean; PRR025, lower bound of the 95% confidence interval for PRR; IC025, lower bound of the 95% confidence interval for IC; EBGM05, lower bound of the 90% confidence interval for EBGM.

A total of 58 unique ICSRs were identified from the JADER dataset. Pemafibrate was involved in 28 drug–event pairs, fenofibrate in 20 pairs, and bezafibrate in 13 pairs (Figure 2). For all fibrates combined, the ROR was 3.74 (95% CI, 2.88–4.85; P < 0.01), PRR was 3.71 (95% CI, 2.86–4.81; χ^2^ > 4), IC was 1.80 (95% CI, 1.42–2.19), and EBGM was 3.35 (90% CI, 2.92–3.83), with all metrics indicating statistically significant associations. By agent, pemafibrate and fenofibrate showed significant signals across all metrics, whereas no significant signals were observed for bezafibrate.

A total of 260 unique ICSRs were identified from the FAERS dataset. Pemafibrate was involved in 3 drug–event pairs, fenofibrate in 233 pairs, and bezafibrate in 25 pairs. For all fibrates, the ROR was 3.81 (95% CI, 3.37–4.31; P < 0.01), PRR was 3.79 (95% CI, 3.36–4.28; χ^2^ > 4), and IC was 1.91 (95% CI, 1.79–2.04), with all metrics showing statistical significance. Signals for ROR and PRR were observed for pemafibrate, while fenofibrate and bezafibrate showed significant signals for ROR, PRR, and IC.

In a sensitivity analysis restricted to primary suspect drugs using the JADER dataset, statistically significant disproportionality was observed for all fibrates combined (N = 36; ROR 10.44 [95% CI, 7.48–14.58], PRR 10.19 [95% CI, 7.37–14.10], IC 3.01 [95% CI, 2.53–3.50]). By agent, the signal was retained for pemafibrate and fenofibrate but not for bezafibrate. In FAERS, all fibrates combined also showed statistically significant disproportionality (N = 67; ROR 6.28 [95% CI, 4.94–8.00], PRR 6.22 [95% CI, 4.90–7.89], IC 2.53 [95% CI, 2.18–2.88]). By agent, the signal was retained for fenofibrate and not retained for bezafibrate; pemafibrate had no primary suspect cases (N = 0), so signal estimation was not applicable.

The detailed results are summarized in Supplementary Tables 2, 3.

### 3.3 Stratified disproportionality analysis

**FIGURE 3:**
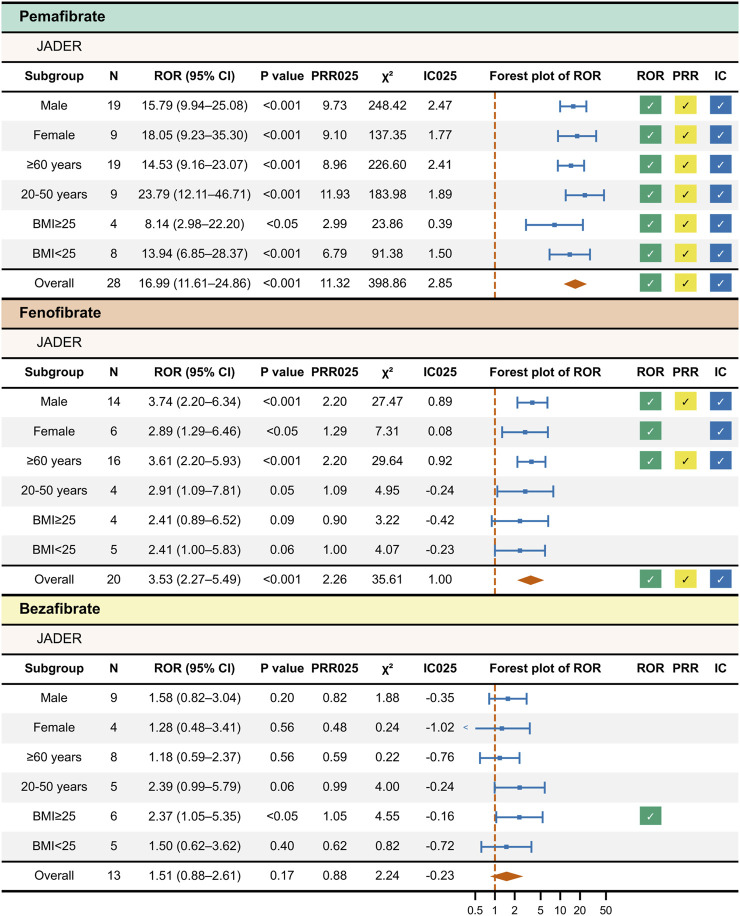
Results of Stratified Disproportionality Analysis of JADER Data. This figure presents the results of stratified disproportionality analysis for each fibrate in the JADER database. Drug–event pairs were extracted for each subgroup—sex (male and female), age (≥60 years and 20–50 years), and BMI (≥25 and <25)—and disproportionality analysis was performed separately for each. A check mark indicates a statistically significant signal.

For pemafibrate, significant ROR, PRR, and IC signals were detected in all strata (Figure 3). For fenofibrate, significant ROR and IC signals were found in the male, female, and age ≥60 years strata, whereas PRR was significant only in the male and age ≥60 years strata. No significant signals were detected for fenofibrate in BMI-based subgroups. For bezafibrate, a significant ROR signal was observed only in the BMI ≥25 stratum. No other significant signals were detected. The detailed stratified results are provided in Supplementary Table 4.

### 3.4 Time-to-onset analysis

In the JADER dataset, TTO data were available only for 60.7% of the pemafibrate ICSRs (N = 17). The events in question comprised nine cases of cholelithiasis, four of cholecystitis, and four of acute cholecystitis (Table 3).

**TABLE 3:** Estimated weibull parameters from time-to-onset analysis.

PTs	n	β	α	Median
Cholelithiasis	9	2.05 [1.61–4.76]	485.93 [322.94–630.29]	525.0 [183.0–706.0]
Cholecystitis/Cholecystitis acute	8	1.29 [0.87–2.94]	447.52 [223.36–705.30]	345.5 [149.0–706.0]
Overall	17	1.59 [1.17–2.56]	469.66 [327.34–613.50]	450.0 [185.0–587.0]

Estimated Weibull parameters and median TTOs for pemafibrate in the JADER dataset (N = 17). This table presents the estimates for each adverse event (cholelithiasis; cholecystitis and cholecystitis acute; all events combined). Values in parentheses indicate 95% CIs (bootstrap).

PTs, Preferred Terms; β, shape parameter; α, scale parameter; TTO, time-to-onset; JADER, Japanese Adverse Drug–Event Report; CI, confidence interval.

**FIGURE 4:**
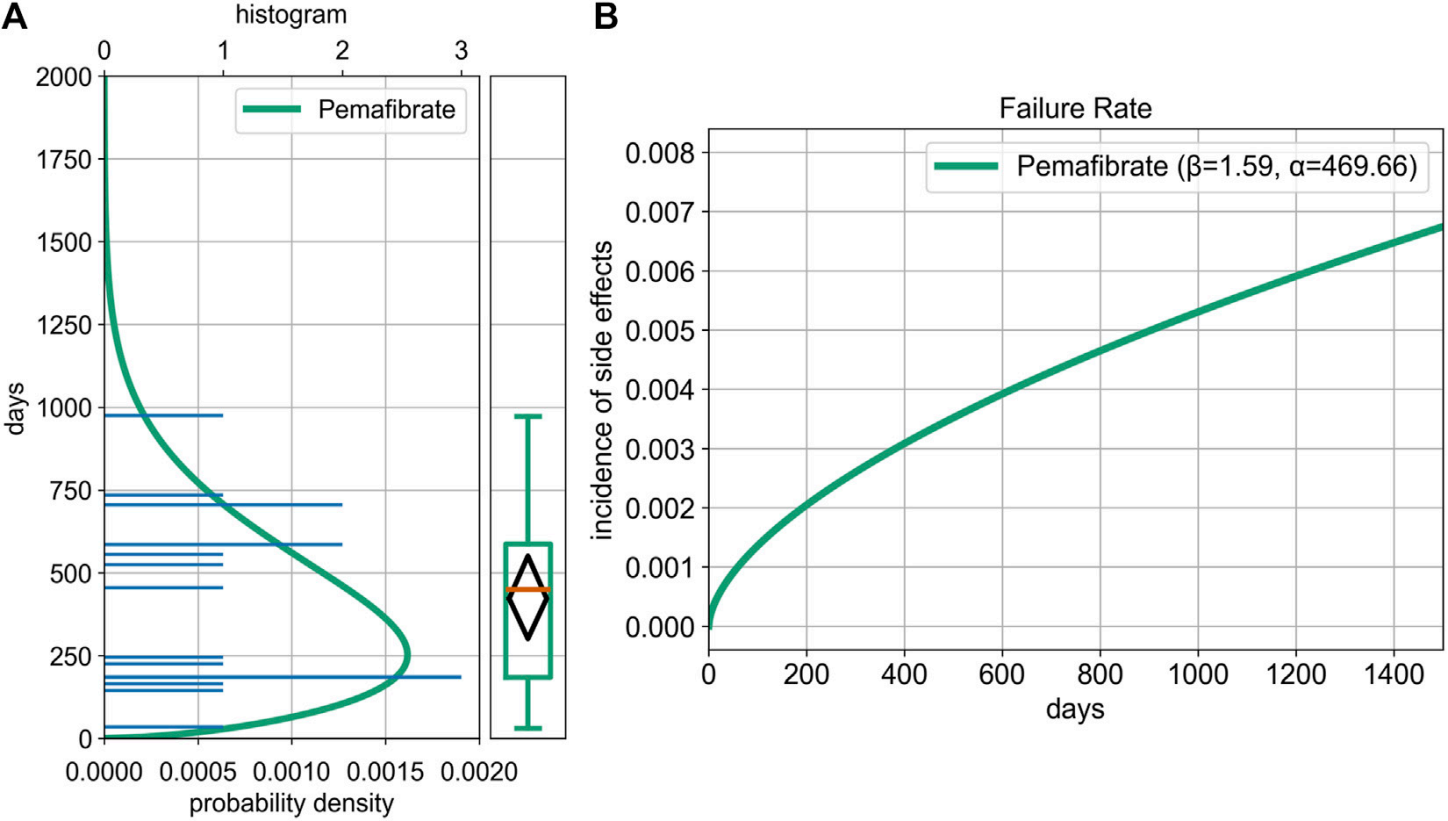
Weibull Distribution and Box Plot Based on Time-to-Onset Analysis of Cholecystitis and Cholelithiasis. Time-to-onset analysis for pemafibrate in the JADER dataset (N = 17); complete date reports only. Median Interquartile Range are shown; shaded band = 95% CI for the Weibull shape parameter (β). **(A)** Histogram, Weibull distribution curve, and box plot based on time-to-onset (TTO) data. The histogram was created using the number of ICSRs, and the Weibull distribution curve was overlaid. **(B)** Failure rate distribution. The Weibull shape parameter (β) exceeded 1, indicating an increasing failure rate over time—a wear-out failure type.

For all cases: α = 469.66 (95% CI, 327.34–613.50), β = 1.59 (95% CI, 1.17–2.56) (Figure 4). The median TTO was 450.0 days (95% CI, 185.0–587.0), with an interquartile range of 185.0–587.0 days.

For cholelithiasis: α = 485.93 (95% CI, 322.94–630.29), β = 2.05 (95% CI, 1.61–4.76). The median TTO was 525.0 days (95% CI, 183.0–706.0).

For cholecystitis and acute cholecystitis: α = 447.52 (95% CI, 223.36–705.30), β = 1.29 (95% CI, 0.87–2.94). The median TTO was 345.5 days (95% CI, 149.0–706.0).

## 4 Discussion

### 4.1 Basic characteristics of the individual case safety reports

This study analyzed preferred terms for cholecystitis and cholelithiasis.

Cholelithiasis is commonly associated with the “5 Fs”—Fair, Fat, Female, Fertile, and Forty—reflecting the higher prevalence encountered in middle-aged women (Bass et al., 2013). Because cholecystitis often arises from gallstones, the two conditions likely share risk factors, although the degree of overlap remains uncertain.

In the JADER dataset, male ICSRs exceeded female ICSRs, contrasting with the known epidemiology of gallstone disease, whereas the skew toward older ages was consistent (Bass et al., 2013). Possible explanations include prescribing patterns—triglyceride-lowering drugs are more often prescribed to men and middle-aged or older adults in Japan (Ministry of Health, Labour and Welfare, 2025)—and reporting bias, where physicians may be more likely to suspect a drug-related cause in men. Similarly, in National Database open data, fibrate prescriptions are concentrated in these age groups and are more commonly prescribed to men (Ministry of Health, Labour and Welfare, 2025). Note that the number of cases represents the number of prescriptions, not the number of patients.

Differences in comorbidity profiles may also contribute to this trend. Metabolic syndrome and type 2 diabetes, both established risk factors for gallstones (Méndez-Sánchez et al., 2005; Aune and Vatten, 2016), are more prevalent in Japanese men (Ministry of Health, Labour and Welfare, 2025). Given that comorbidity data in JADER are often incomplete, population-based cohort studies including both drug-exposed and unexposed individuals are needed to distinguish drug effects from metabolic risk.

### 4.2 Disproportionality analyses

Primary disproportionality analysis conducted on the JADER dataset detected clear biliary tract disease signals for fibrates. Pemafibrate showed significant disproportionality across ROR, PRR, IC, and EBGM, with stratified analysis confirming consistent signals in all subgroups. These findings suggest an association between fibrates and biliary ADEs.

In the JADER dataset, consistent signals were observed for pemafibrate, while fewer signals were detected for fenofibrate and bezafibrate, respectively. The presence or absence of such signals may reflect not only pharmacological characteristics, such as PPARα selectivity, but also differences in patient characteristics, including demographic, genetic, and metabolic factors.

Pemafibrate has a markedly higher selectivity for PPARα than other fibrates while retaining strong triglyceride-lowering effects (Honda et al., 2022). PPARα agonism can lower bile acid output by reducing CYP7A1 activity and CYP27A1 mRNA levels (Post et al., 2001; Ghonem et al., 2015). Mechanistically, PPARα agonists diminish hepatocyte nuclear factor-4α binding to the direct repeat 1 element on the CYP7A1 promoter, and PPARα may antagonize liver X receptor-dependent activation of CYP7A1 (Marrapodi and Chiang, 2000; Rakhshandehroo et al., 2010). Beyond effects on total output, bile acid composition is partly governed by sterol 12-alpha-hydroxylase (CYP8B1). PPARα activation increases hepatic CYP8B1, thereby raising 12α-hydroxylated bile acids and the cholic acid/chenodeoxycholic acid ratio (Chiang, 2004; Xie et al., 2019). Because cholic acid-rich bile is generally more cholesterol-saturated than chenodeoxycholic acid-rich bile, this shift may increase the cholesterol saturation index and the risk of cholesterol gallstones (Einarsson and Grundy, 1980). Given its high selectivity for PPARα, pemafibrate may amplify these pathways and alter bile acid homeostasis, potentially increasing gallstone risk.

Further research is needed to determine whether this represents a class effect, a drug-specific phenomenon, or an interaction with patient risk factors. Mechanistic *in vivo* and *in vitro* studies could clarify how pemafibrate alters bile acid synthesis and cholesterol saturation. Large population-based cohorts are also necessary to quantify risk and adjust for confounding factors. Integrating pharmacovigilance data with observational and mechanistic studies will be essential for evidence-based prescribing and monitoring.

In the United States (U.S.), adult obesity (BMI ≥30: 41.9%) exceeds that in Japan (BMI ≥25: 25.3%; ≥30: 4.5%) (Stierman et al., 2021; Ministry of Health, Labour and Welfare, 2025), indicating a higher baseline risk of gallstones in the U.S. Even so, a signal was detected in Japan’s JADER database, suggesting drug-related contributions to reported gallstone disease and cholecystitis. Cross-country differences in prescribing likely influenced signal detection. In Japan, fenofibrate and bezafibrate have long been used, and pemafibrate use has increased since its approval. In the U.S., fenofibrate predominates, whereas bezafibrate and pemafibrate are not approved (Jackevicius et al., 2011; Arai et al., 2024). FAERS reporting is U.S.-centric, and the case counts (N) in the primary analysis likely reflect this exposure distribution.

### 4.3 Time-to-onset analysis

TTO analysis helps identify temporal patterns in the development of ADEs (Leroy et al., 2014). Because gallstones may remain asymptomatic for over 2 years (Mok et al., 1986), TTO in SRSs often reflects diagnosis or symptom onset rather than true onset (Leroy et al., 2014).

In this study, Weibull analysis suggested an increasing risk of biliary ADEs with prolonged pemafibrate use. The median TTO was 450 days (95% CI, 185.0–587.0), which is shorter than that reported in previous studies (Mok et al., 1986), possibly owing to fibrate pharmacology and early-reporting bias in SRSs (Goldman, 1998).

Seven cholecystitis cases were identified (four acute, three unspecified). The acute cases showed a wide TTO range (31–706 days), with the upper bound unusually high for an acute presentation. Most acute cholecystitis cases result from cystic duct obstruction by gallstones (Gallaher and Charles, 2022), suggesting that gallstone formation may have preceded inflammation. However, the limited clinical data in SRSs precludes causal inferences. Given that pemafibrate is contraindicated in patients with cholelithiasis (Kowa Co.ltd., 2024) and often discontinued after biliary ADEs, regular hepatobiliary monitoring, including gallstone assessment, is recommended. Based on the median TTO of about 450 days in this study, starting hepatobiliary monitoring around 6 months after treatment begins and continuing it at least once a year seems reasonable. Monitoring should include checking for biliary symptoms, liver function tests, and abdominal ultrasonography when needed. These findings should be interpreted with caution because the Weibull analysis was based on a small number of cases. More work is needed to better define the optimal timing, frequency, and approach to monitoring.

While caution is warranted regarding the potential biliary risks associated with fibrate-class drugs, these agents have demonstrated cardiovascular benefits in patients with dyslipidemia (Jun et al., 2010). Therefore, clinicians should carefully weigh the cardiovascular benefits against the biliary risks and tailor treatment and monitoring strategies to individual patients.

### 4.4 Case in clinical practice

**FIGURE 5:**
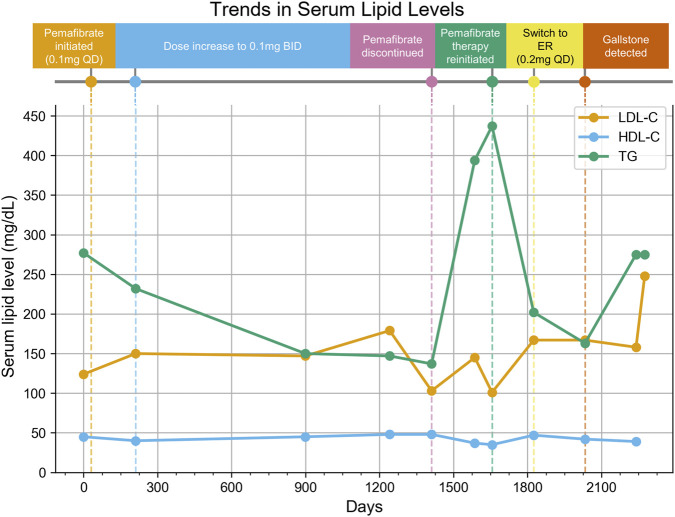
Treatment Timeline and Trends in Serum Lipid Levels. Vertical dashed lines indicate major clinical events during the treatment course, and the lines represent LDL-C (orange), HDL-C (blue), and TG (green) levels at each measurement point. The x-axis shows the date, and the y-axis shows serum lipid levels (mg/dL). QD: quaque die (once daily), BID: bis in die (twice daily), ER: extended-release, LDL-C: Low-Density Lipoprotein Cholesterol, HDL-C: High-Density Lipoprotein Cholesterol, TG: Triglycerides.

We report a real-world case of gallstone detection after long-term pemafibrate use in an elderly Japanese woman (Figure 5). She switched from ezetimibe to pemafibrate at an initial daily dosage of 0.1 mg, which was subsequently increased to 0.2 mg daily. The treatment was stopped after 3 years and 10 months after her lipid levels improved. The treatment was resumed 8 months later using the extended-release tablet.

One year after resuming treatment, a routine ultrasound revealed a single small gallstone. She had no history of cholelithiasis and was asymptomatic. The only risk factors identified were female sex and age >40 years. The total duration of pemafibrate treatment was approximately 1,760 days.

Although causality is unproven, a pemafibrate-associated ADE cannot be excluded as a possible etiology of the patient’s condition. Our SRS analysis found consistent, significant biliary ADE signals across sex, age, and BMI strata. This case is clinically consistent with those findings and highlights the need to reassess biliary ADE risk with long-term fibrate use. In elderly patients, gallstones often remain asymptomatic; therefore, regular imaging-based monitoring should be considered regardless of symptoms.

### 4.5 Limitations

Although SRSs are useful for early signal detection and post-marketing surveillance (Montastruc et al., 2006), they have some limitations (Kasliwal, 2012; Noguchi et al., 2021). Underreporting is substantial, with a median rate of nearly 94% (Hazell and Shakir, 2006). Reporting bias is common, with peaks observed soon after drug approval (Weber effect) (Weber, 1987; Raschi et al., 2013) and increases in reporting following label warnings or publications (notoriety bias) (Pariente et al., 2007). Pemafibrate was launched in Japan in 2018, and its safety data may have been influenced by the Weber effect. However, continuous reporting of cholecystitis and cholelithiasis was observed without early clustering, suggesting only a limited impact of the Weber effect. Nevertheless, its potential influence cannot be completely excluded due to the inherent characteristics of SRS data. Severe or novel events also tend to be selectively reported (Hazell and Shakir, 2006; Matsuda et al., 2015). Data on key patient characteristics are often missing (Dang et al., 2022), which limits adjustment for confounding factors. Furthermore, known gallstone risk factors (obesity, female sex, and diabetes) were more frequent among patients treated with fibrates, and this imbalance may have contributed to the observed signals. Incidence rates cannot be calculated because only patients with ADEs are included (Goldman, 1998).

These sources of bias and confounding factors cannot be adequately controlled in SRS analyses. Therefore, findings should be interpreted as indicators of disproportional reporting rather than evidence of causality. For some drug–event combinations in this study, the number of cases was small, resulting in limited statistical power and wide uncertainty in the estimates. The time-to-onset assessment relied on only 17 cases with complete date information and did not account for the effects of treatment discontinuation or dose adjustments. Consequently, these results should be regarded as hypothesis-generating and require confirmation in larger, well-designed studies.

## 5 Conclusion

This study suggests an association between fibrates and biliary ADEs, particularly cholecystitis and cholelithiasis.

These findings provide valuable insights to guide future mechanistic and epidemiological studies. In clinical practice, careful attention should be paid to biliary ADEs during fibrate therapy. Regular hepatobiliary monitoring should be tailored to the patient’s background and maintained throughout treatment.

## Data Availability

Publicly available datasets were analyzed in this study. This data can be found here: JADER data are available from the PMDA website (https://www.pmda.go.jp/safety/info-services/drugs/adr-info/suspected-adr/0003.html) FAERS data are available from the FDA website (https://fis.fda.gov/extensions/FPD-QDE-FAERS/FPD-QDE-FAERS.html).
