# Supplementary material for: Disproportionality analysis of biliary adverse events associated with fibrates using the JADER and FAERS databases
Source: Front Pharmacol. 2025 Nov 24;16:1700589. doi: 10.3389/fphar.2025.1700589 (PMC12684286; doi:10.3389/fphar.2025.1700589)
Supplement: Supplementary file 3 [file DataSheet1.pdf]

Supplementary Material

Supplementary Methods 1

S1.1 Reporting Odds Ratio (ROR).

ROR is a sensitive screening metric and the standard indicator used in JADER (Sakaeda *et al.*, 2013). A signal was considered present when $n_{11} \geq 3$ and the lower bound of the 95% confidence interval (ROR025) exceeded 1. P values were computed using Fisher's exact test.

$$ROR = \frac{n_{11}/n_{21}}{n_{12}/n_{22}}$$

$$95\%CI = \exp\left\{\log(ROR) \pm 1.96\sqrt{\frac{1}{n_{11}} + \frac{1}{n_{12}} + \frac{1}{n_{21}} + \frac{1}{n_{22}}}\right\}$$

S1.2 Proportional Reporting Ratio (PRR).

PRR compares the proportion of a specific adverse event for a given drug with that for all other drugs and follows the implementation of the Medicines and Healthcare products Regulatory Agency in the UK (Evans *et al*., 2001). In this study, the lower bound of the 95% confidence interval for PRR (PRR025) was used to more strictly evaluate drugs that had already passed initial screening based on ROR. While this higher threshold can reduce false positives, it may also increase the risk of missing important signals (Harpaz *et al*., 2013). To assess the statistical significance of the signal, we calculated the chi-square (χ^2) statistic using the chi-square test. A statistically significant signal was defined as one in which n_{11} was ≥ 3, PRR025 exceeded 2, and the χ^2 value exceeded 4.

$$PRR = \frac{n_{11}/n_{1+}}{n_{21}/n_{2+}}$$

$$95\%CI = \exp\left\{\log(PRR) \pm 1.96\sqrt{\frac{1}{n_{11}} - \frac{1}{n_{1+}} + \frac{1}{n_{21}} - \frac{1}{n_{2+}}}\right\}$$

S1.3 Bayesian confidence propagation neural network (BCPNN) and Information Component (IC).

BCPNN is a Bayesian approach implemented as a standard signal-detection algorithm in VigiBase, the World Health Organization global database (Orre *et al.*, 2000). It is robust for sparse data through the use of prior distributions, which stabilize estimates (Bate *et al.*, 1998).

$$E(IC_{11}) = \log_2 \frac{(n_{11} + \gamma_{11})(n_{++} + \alpha)(n_{++} + \beta)}{(n_{++} + \gamma)(n_{1+} + \alpha_1)(n_{+1} + \beta_1)}$$

$$V(IC_{11}) = \left(\frac{1}{\log 2}\right)^2 \left[\frac{n_{++} - n_{11} + \gamma - \gamma_{11}}{(n_{11} + \gamma_{11})(1 + n_{++} + \gamma)} + \frac{n_{++} - n_{+1} + \alpha - \alpha_1}{(n_{1+} + \alpha_1)(1 + n_{++} + \alpha)} + \frac{n_{++} - n_{+1} + \beta - \beta_1}{(n_{+1} + \beta_1)(1 + n_{++} + \beta)}\right]$$

$$\gamma = \gamma_{11} \frac{(n_{++}+\alpha)(n_{++}+\beta)}{(n_{1+}+\alpha_1)(n_{+1}+\beta_1)} \quad \gamma_{11} = 1 \quad \alpha_1 = \beta_1 = 1 \quad \alpha = \beta = 2$$

$$95\%CI = E\left(IC_{11} \pm 2\sqrt{V(IC_{11})}\right)$$

Based on the formula above, we calculated the information component (IC). Signals were determined using the lower bound of the 95% confidence interval (IC025). A signal was considered present when IC025, the lower bound of the 95% credibility interval, exceeded 0.

S1.4 Gamma–Poisson Shrinker (GPS) and EBGM.

We adopted a Bayesian estimation approach with a two-component gamma-mixture prior to compute the empirical Bayes geometric mean (EBGM) (Dumouchel, 1999). The Gamma–Poisson Shrinker was previously used by the FDA; the Multi-Item Gamma–Poisson Shrinker, an extension for detecting drug interactions, is used currently (Szarfman *et al.*, 2002). A key advantage is the ability to adjust for confounding via stratification (Szarfman *et al*., 2004). The observed count (*O*) for each drug–ADE pair was assumed to follow a Poisson distribution. The expected count (*E*) was computed under the independence model; within stratum *s*, n_s denotes the total number of reports and P_i and P_j the reporting proportions for drug *i* and event *j*. We calculated EBGM as the exponential of the posterior expectation of log λ; the 90% CI was obtained from the 5th and 95th percentiles of the posterior distribution, and a signal was considered present when the lower bound (EBGM05) was ≥ 2.0. Because stratified disproportionality analysis was not performed for FAERS, EBGM was not computed for that database.

$$E_{ij} = \sum_s n_s \cdot P_i \cdot P_j$$
